# The New Hybrid Nutrient Density Score NRFh 4:3:3 Tested in Relation to Affordable Nutrient Density and Healthy Eating Index 2015: Analyses of NHANES Data 2013–16

**DOI:** 10.3390/nu13051734

**Published:** 2021-05-20

**Authors:** Adam Drewnowski, Jessica Smith, Victor L. Fulgoni

**Affiliations:** 1 Center for Public Health Nutrition, University of Washington, Box 353410, Seattle, WA 98195, USA; 2 General Mills Scientific and Regulatory Affairs, Minneapolis, MN 55426, USA; Jessica.Smith@genmills.com; 3 Nutrition Impact LLC, Battle Creek, MI 49014, USA; vic3rd@aol.com

**Keywords:** nutrient profiling, hybrid nutrient density score, whole grain, fruit, dairy, NHANES 2013–16, replacement modeling, food prices, healthy eating index 2015, affordable nutrient density

## Abstract

**Background:** Hybrid nutrient density scores are based on both nutrients and selected food groups. **Objective:** To compare the new hybrid nutrient-rich food NRFh 4:3:3 score to other nutrient-rich food (NRF) scores, energy density, and energy cost and to model the impact on the Healthy Eating Index (HEI-2015) of partially replacing less nutrient-rich with more nutrient-rich foods. **Methods:** Analyses were based on 5870 foods and beverages in the Food and Nutrient Database for Dietary Studies and on 24 h dietary recalls from the National Health and Nutrition Examination Survey (NHANES 2013–16). The NRFh 4:3:3 model was based on four nutrients to encourage (protein fiber, potassium, MUFA + PUFA); three food groups to encourage (dairy, fruit, whole grains); and three nutrients to limit (saturated fat, added sugar, sodium). Ratings generated by NRFh 4:3:3 and by other NRF models were correlated with score components, energy density (kcal/100 g), and energy cost (USD/100 kcal). The impact on HEI-2015 of replacing foods in the lowest nutrient density tertile (T1) with top tertile (T3) foods at 10%, 20%, 30%, and 100% equicaloric replacement was modeled using NHANES 2013–16 dietary data by population subgroups. **Results:** The NRFh 4:3:3 model awarded higher scores to foods containing dairy, fruit, and whole grains and proportionately lower scores to vegetables when compared to the NRF 9.3 model. Higher NRF and NRFh nutrient density scores were linked to lower energy density and higher energy cost; however, both correlations were lower for the NRFh 4:3:3. Isocaloric replacement of bottom tertile with top tertile foods as rated by both models led to significantly higher HEI-2105 values, based on complete (100%) and on partial (10–30%) replacement. **Conclusion:** The new NRFh 4:3:3 model provides the basis for developing new metrics of affordable nutrient density. The model identified “best value” food categories that were both affordable and nutrient-rich. Total and partial replacement of low nutrient density with high nutrient density foods was associated with higher HEI-2015 scores, suggesting that even partial inclusion of more nutrient dense foods in the diet may have an important impact on total diet quality.

## 1. Introduction

*Dietary Guidelines for Americans* have long stressed the importance of healthy food patterns and healthy food groups [1,2,3]. Most nutrient profiling (NP) models, designed to score nutrient density of foods, base their scores on the food’s energy content and nutrient composition [4,5,6]. The intent of dietary guidelines would be better served by hybrid NP models that combine nutrients with selected food groups [7,8]. The new NRFh 4:3:3 score, based on nutrients and on three food groups (whole grains, dairy, and fruit) may better capture a food’s overall nutritional value and its place in healthy food patterns.

The goal of nutrient profiling is to capture a food’s nutritional value. The new hybrid NRFh models need to be compared to the established ones [9,10] and also tested in relation to the foods’ energy density, defined as kcal/100 g. In past studies [11,12], the preference was for those profiling algorithms that showed low correlations between nutrient density scores and energy density of foods. Those NP models that score calories, total sugar, and saturated fat tend to capture the foods’ energy density as opposed to their nutrient content [13,14,15]. Second, it is important to ensure that nutrient density scores generated by NP models are not correlated too highly with food prices, also expressed per 100 kcal of food [11,16]. The principal objective is not to point to higher-cost foods but to identify those foods that are both nutrient-rich and affordable [4,14].

The inclusion of food groups in nutrient profiling will help align nutrient density metrics with the current dietary guidelines, both in the US and elsewhere [7,8]. One example is provided by whole grains [13]. Included as a key component of dietary guidelines worldwide [1,2,3,13], whole grains are not a part of most nutrient density metrics. A similar case can be made for including fruit and low-fat dairy in nutrient profiling schemes [17]. New NP models need to incorporate MyPlate food groups together with nutrients of public health concern, without forgetting about the need to limit saturated fat, added sugar, and salt.

The present NRFh 4:3:3 model is one of the few to include whole grains, fruit, and dairy alongside nutrients to encourage and nutrients to limit [8]. This study compared scores generated by the NRFh 4:3:3 model to scores derived using other nutrient-rich food models and tested model performance in relation to energy density and energy cost. NHANES 2013–16 data were used to determine whether replacing lower-scoring with higher-scoring foods, based on nutrient density tertiles, would lead to improved HEI-2015 scores, a measure of adherence to dietary guidelines.

## 2. Materials and Methods

### 2.1. Data Source and Population

Data analyses were based the What We Eat In America dietary assessment component of two consecutive cycles of the nationally representative cross-sectional National Health and Nutrition Examination Survey (NHANES) for years 2013–2014 and 2015–2016 [18]. The NHANES is the main source of dietary surveillance data in the US and serves to inform the *Dietary Guidelines for Americans* and other federal and state food and nutrition policies [18]. The dietary recall component uses a multi-pass method and measures all foods consumed midnight-to-midnight during the day prior to data collection [18]. The present analyses were based on 15,781 participants aged ≥2 years who completed a valid 24 h recall, as defined by National Center for Health Statistics staff. All analyses were adjusted for the complex sample design of NHANES. For analytical purposes, dietary intakes data were stratified by gender (male, female) age group (2–18, >18 years) and by poverty-to-income-ratio or PIR (cutpoints: <1.35, 1.35 to 1.85, and >1.85).

### 2.2. Food and Nutrient Database for Dietary Studies (FNDDS)

The Food and Nutrient Database for Dietary Studies (FNDDS) maintained by the US Department of Agriculture was used to calculate energy and nutrient content of foods consumed by NHANES participants [19]. For the present analyses, foods and beverages with energy density of <10 kcal/100 g were excluded (water, diet soft drinks, unsweetened coffee and tea). Alcoholic beverages, baby foods and infant formula, non-reconstituted nutrition powders, and items not classified as foods were also excluded. Analyses were thus based on 5870 foods and beverages.

The 5870 foods were aggregated into multiple food groups, subgroups, and categories using What We Eat in America coding schemes [19]. The one-digit codes identify 9 major food groups: milk and milk products; meat, poultry, and fish; eggs; dry beans and legumes; grains; fruits; vegetables; fats and oils; and sugars, sweets, and beverages. The two-digit codes identify 53 smaller food subgroups. Foods in the dairy group are now separated into milks and yogurts, creams, dairy desserts, and cheeses. The meat group is separated into beef, pork, lamb, poultry, organ and processed meats, fish and shellfish, mixed meat dishes, and soups. The four-digit codes identify 138 food categories. The eight-digit codes correspond to the individual foods (*N* = 5870).

### 2.3. The USDA National Food Prices Database

The original national food prices database, first released by the US Department of Agriculture in 2008, provided retail food and beverage prices for all foods consumed by NHANES 2003–2004 participants. The prices were expressed in dollars per 100 g, edible portion, adjusting for preparation losses and gains [20]. The 2001–04 prices were adjusted for inflation to the period 2013–2016 as described previously [21]. Briefly the 2001–2004 FNDDS food codes were mapped to items listed in the Consumer Price Index from the Bureau of Labor Statistics [21]. Any new FNDDS 2013–2016 food codes were matched with the corresponding food codes in the FNDDS 2001–2004. Mixed dishes that did not map to a single Bureau of Labor Statistics series were regressed on the Food Patterns Equivalents Database [21]. The regression coefficients were then applied to product recipes. Monthly food prices were averaged over the 2 years of NHANES cycles.

### 2.4. Nutrient Profiling (NP) Methods

Nutrient density of foods can be calculated per 100 kcal (standard approach) or per serving size [15]. In the US, serving sizes for nutrition labeling are known as the “Reference Amounts Customarily Consumed” and are mandated by the Food and Drug Administration [22]. Scores for nutrients and food groups in the nutrient-rich food models were calculated as a percentage of daily recommendations. For nutrients, the Food and Drug Administration daily values were used as the reference amounts of a nutrient to consume or not exceed per day for the population 4 years and older [15]. For food groups, recommended levels of dairy, whole grains, and fruit were based on *Dietary Guidelines for Americans*, and specifically the Healthy US-Style Dietary Pattern at the 2000 kcal level (3). These values are summarized in Table 1.

### 2.5. The NRF n.3 Family of Scores

The nutrient-rich foods (NRF n.3) index is based on two sub-scores: NRn and LIM. The positive NRn sub-score is based on a variable number n of nutrients to encourage, whereas the negative LIM sub-score is based on the same 3 nutrients to limit (saturated fat, added sugars, and sodium). Both sub-scores NRn and LIM are calculated as the sum of percent daily value per 100 kcal of food [23]. As in past calculations, percent daily values for nutrients were truncated at 100% [23]. The NRF n.3 scores were then calculated by subtracting LIM from NRn scores. The final NRF n.3 algorithms are given by NRF n.3 = NRn − LIM. The present analyses used multiple versions of the NRF score that were based on 4, 6, 8, and 9 nutrients to encourage, respectively. The score components are listed in Table 2.

### 2.6. Hybrid NRFh 4:3:3 Score

The NRFh model was developed following a total of 2,162,720 iterative regression analyses against HEI-2015 diet quality scores [8]. Models based on 16 nutrients explained 66% of the variance, whereas those based on 5 MyPlate food groups explained 50%. The NRFh 4.3.3 model, based on nutrients and food groups, explained 72% [8].

The NRFh 4.3.3 model was based on four nutrients to encourage (protein, fiber, potassium, and dietary MUFA + PUFA); three food groups to encourage (whole grains, dairy, and fruit); and three nutrients to limit (saturated fat, sodium, and added sugar). The overall model structure followed the framework NRFh = 100 × (NRx + MPz − LIMy).

### 2.7. Replacement Modeling Approach

All foods and beverages that were available for NRF replacement modeling [23,24,25,26] were identified in the individual foods data file in the NHANES 2013–16. First, tertile cut-points for both NRF scores (NRF9.3 and NRFh 4:3:3) were based on food categories as identified by What We Eat in America codes and were population-specific, given the diversity of dietary patterns across population strata. Separate tertiles were established for subgroups by age, gender, race/ethnicity, and poverty-to-income ratio. Thus, replacement modeling was based on consumption patterns for 12 groups stratified by age and gender (6 age groups (2–18, 2–8, 9–18, 19–99, 19–59, and 51–99 years) × 2 genders); and 36 groups stratified by race/ethnicity and poverty-to-income ratio category (3 age groups (19–99, 19–59, 51–99 years) × 2 genders × 6 demographic subgroups (Hispanic, Black, Asian, PIR Low, PIR Med, PIR High). Presented here are analyses by age and PIR, as these findings were most relevant to the concept of affordable nutrient density.

Replacement modeling was based on weighted composite NRF nutrient density scores. The weighted composite nutrient density of foods per 100 kcal in the lowest tertile of nutrient density (T1) and in the highest tertile (T3) was determined by weighting the foods’ NRF scores by the amounts eaten. Using population-specific tertiles, foods with nutrient density scores below the T1 cut-point were assigned to the T1 category. All foods with nutrient density scores above the T3 cut-point were assigned to the T3 category. Weighted composite nutrient profiles for foods in T1 and T3 categories were used in replacement modeling.

Replacement modeling was equicaloric. For each participant, T1 foods were replaced by equivalent calories (but different nutrients and food group amounts) from T3 foods. The four models used 10%, 20%, 30%, or 100% energy replacement, respectively. In each case, percent of energy from T1 foods was replaced by an equal amount of energy from T3 foods. For example, for the 10% energy replacement, 10% of composite of T1 foods was removed from that participant’s diet and replaced with an equivalent amount of calories (and nutrients/food groups) from a composite of T3 foods. Foods with low energy density (<10 kcal/100 g) were included in replacement modeling but were not used in determining tertile cutoffs.

HEI-2015 values were calculated following each replacement analysis and delta HEI-2015 values obtained for each participant. The method was similar to a paired t-test, with the *p*-value testing the hypothesis that the mean change in HEI-2015 was zero.

## 3. Results

### 3.1. Relation Between NRFh 4:3:3 and NRF 9.3 Scores

**Figure 1:**
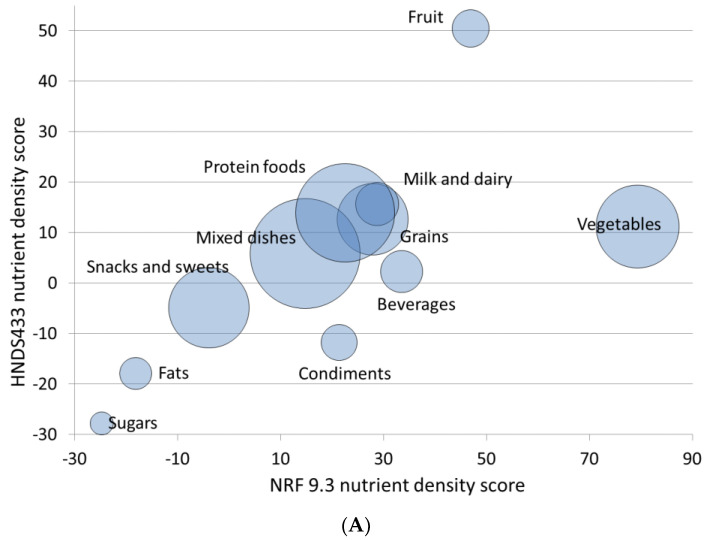

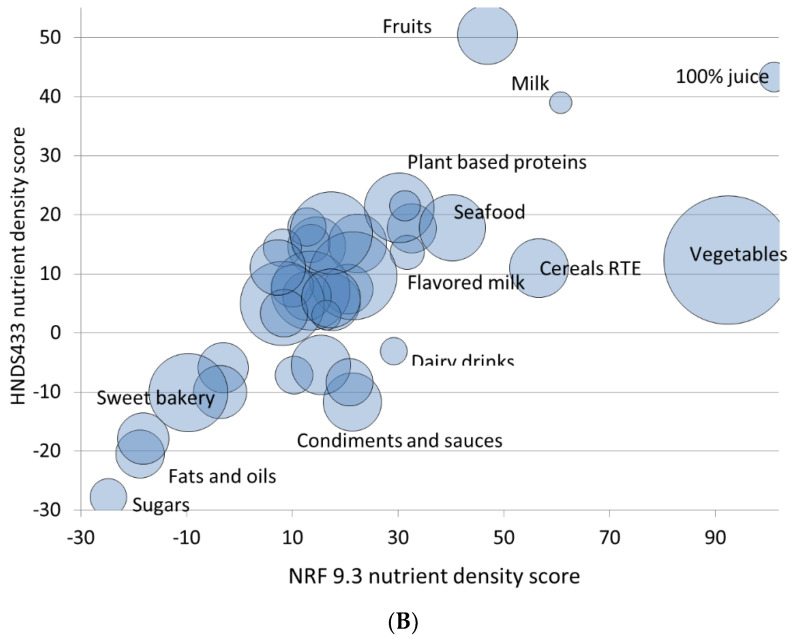
Relation between NRF 9.3 score and NRFh 4:3:3 by (**A**) (**top panel**) WWEIA food group and (**B**) (**bottom panel**) WWEIA food category. Size of the bubble denotes the number of foods within each food group or category.

Figure 1 shows the relation between NRF 9.3 and NRFh 4:3:3 by food group (1A) or by food category (1B). The two models were correlated (r = 0.44). However, systematic differences were observed. Figure 1A,B shows that the hybrid NRFh 4:3:3 model gave higher ratings to fruit, dairy, and plant-based proteins and proportionately lower ratings to vegetables as compared to the NRF9.3 model

### 3.2. Relation Between NRFh 4:3:3 Score and Score Components

Table 3 shows correlations among nutrient density scores, energy density of foods, and individual nutrient daily values. First, all nutrient density scores were negatively correlated with energy density. The correlation was highest for the NRF 9.3 score and progressively diminished for those NRF models that had fewer nutrients. The correlation between NRFh 4.3.3 scores and energy density was −0.19, which was attenuated when compared to the NRF9.3 model but still significant. Significant correlations between the NRFh 4:3:3, other NRF scores, and model components, expressed as percent daily values, are also shown. For the NRF 9.3 model, there were significant correlations with model components: protein (r = 0.20), fiber (r = 0.67), vitamin A (r = 0.599), vitamin C (r = 0.734), vitamin D (r = 0.129), calcium (r = 0.539), iron (r = 0.530), potassium (r = 0.718), and magnesium (r = 0.641). The correlation with fruit was significant but not with dairy or whole grains.

The NRFh 4:3:3 scores were correlated less strongly with nutrients and more strongly with food groups (as intended). Correlations with fruit (r = 0.491), whole grains (r = 0.292), and dairy (r = 0.126) were higher relative to the NRF 9.3 model, whereas correlations with fiber (r = 0.35), vitamin C (r = 0.19), calcium (r = 0.192), and potassium (r = 0.327), iron (r = 0.138), and magnesium (r = 0.300), while still significant, were much lower.

Table 3 also shows inverse correlations between NRFh 4:3:3, other NRF scores, and energy cost, calculated per 100 kcal. For all models there was a positive correlation between nutrients per 100 kcal and cost per 100 kcal. That correlation was lower for the NRF models with fewer nutrients. For the NRFh 4.3.3 model, the correlation between nutrient density and cost was attenuated but still significant.

### 3.3. Affordable Nutrient Density

**Figure 2:**
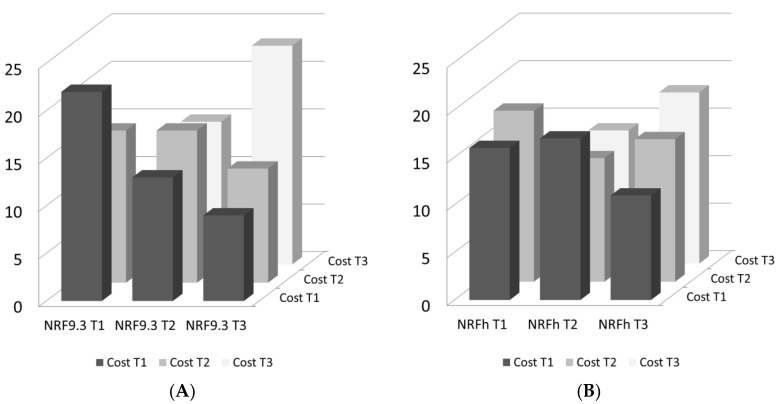
Relation between tertiles of nutrient density per 100 kcal using (**A**) (**right panel**) NRF 9.3 index and (**B**) (**left panel**) NRFh 4:3:3 index and tertiles of cost per 100 kcal.

Figure 2 shows the relation between tertiles of nutrient density and tertiles of cost per 100 kcal. In general, foods in the top nutrient density tertile also tended to be more expensive. It should be noted that the relation between modeled nutrient density and cost was much stronger for the NRF 9.3 model than it was for the NRFh 4:3:3 model. In particular, based on NRF, there was a limited choice of food groups that would simultaneously qualify as both affordable and nutrient dense. That range was expanded when the NRFh 4:3:3 score was the principal nutrient density measure. As shown, foods in the lowest cost tertile were mostly those of lower nutrient density; by contrast, foods in the top tertile of nutrient density were almost always more expensive. This relation was strong for NRF 9.3 but was attenuated with NRFh 4:3:3.

### 3.4. Replacing Low Nutrient Density with High Nutrient Density Foods

Table 4 lists food categories, identified by What We Eat in America four-digit codes by tertile of nutrient density based on NRF 9.3 and NRFh 4.3.3 scores, respectively. The first column shows food categories in the bottom tertile of mean NRF 9.3 scores. The second column shows food categories in the top tertile of NRF 9.3 scores. As expected, food categories in the bottom tertile of NRF9.3 scores were fats and sweets, candy and desserts, sweetened soft drinks, and processed meats. Food categories in the top tertile included milk and dairy products, fish and shellfish, a wide variety of fresh vegetables and fruit, and ready to eat cereals. Consistent with past reports, mean cost per 100 kcal was higher for the more nutrient-rich food categories. Mean cost increased from 0.27 US dollars per 100 kcal to 0.70 US dollars per 100 kcal.

Food categories in the bottom and top tertiles of NRFh 4:3:3 scores are also shown. As previously, food categories in the bottom tertile of NRFh 4:3:3 scores were fats and sweets, candy and desserts, sweetened soft drinks, and processed meats. However, with the new hybrid scoring system, the list of nutrient-rich foods as identified by the NRFh 4:3:3 model was modified to include pasta, noodles, and cooked grains; cheese, bananas, nuts, and seeds; and beans, peas and legumes. Those had been identified previously as affordable nutrient-rich foods. As a result, there was less disparity in per calorie cost between food categories in the bottom tertile and those in the top tertile of NRFh 4:3:3 scores (USD 0.37 to 0.58).

It should be noted that the What We Eat in America four-digit codes identify food categories and not only single foods. The categories include condiments, salad dressings, and dips that are not typically eaten on their own but accompany other food items. Culinary ingredients (fats, sweets, gravies) have low per calorie costs. The inclusion of margarine, mayonnaise, and other culinary ingredients among bottom tertile food categories helped to lower mean per calorie cost.

Table 5 shows the impact on HEI-2015 scores by age group following food replacement based on NRFh 4:3:3 ratings. T1 foods in the diet were replaced with T3 foods at four different levels: 10, 20, 30, and 100%. HEI-2015 scores improved with higher percent replacement for 2–18 and 19+ year age groups. Although maximum improvement was obtained with 100% replacement, significant increases in HEI-2015 scores were also obtained with only partial replacement. When 10% of the T1 foods were replaced with T3 foods, HEI-2105 scores improved by 2.8 to 4.1 points for males and 2–7 to 4.0 for females. Further replacement of 20 and 30% of T1 foods led to an HEI-2015 increase of 4.8 to 6.5 points in males and 4.7 to 6.5 points in females. In adults, significant improvement in HEI-2015 was obtained with 30% replacement based on the NRFh 4:3:3 score.

The impact on HEI-2015 scores by age group following food replacement based on NRF 9.3 tertiles is also shown. The change in HEI-2015 increased as percent replacement increased for all scores in 2–18 and 19+ years. In general, the increase was in the order of 3 to 6 HEI-2015 points. The non-overlapping confidence interval values show that the NRFh 4:3:3 performed better than the NRF 9.3 model.

Table 6 shows increases in delta HEI-2015 stratified by PIR. Again, significant improvements in HEI-2015 were achieved with a 10–30% replacement of T1 foods by T3 foods. The effect was greater for the NRFh 4.3.3 model than for the NRF9.3 model.

## 4. Discussion

The NRFh 4:3:3 was initially developed using a posteriori approach, with elements selected to optimize the correlation between nutrient density scores and HEI-2015, a measure of adherence to Dietary Guidelines. This was a reversal of the conventional profiling paradigm. More often, NP models derived a priori are tested against independent measures of a healthy diet. The NRFh 4:3:3 score derived in this manner was based on seven nutrients and three food groups: dairy, fruit, and whole grains. Mean nutrient density scores showed that score values for foods containing dairy, fruit, and whole grains were elevated compared to NRF 9.3, whereas nutrient density scores for vegetables were correspondingly reduced.

As more NP models are developed, their performance needs to be tested with respect to some recognized standards, such as affordability. Nutrient profile models are based on protein, fiber, vitamins, and minerals and calculated per 100 kcal based their scores on nutrient to calorie ratios. Their algorithms operationalize the FDA definition that nutritious foods should provide “substantial” amounts of desirable nutrients in relation to “few” calories [27]. Not surprisingly, these models were negatively related to energy density and positively related to per calorie cost [11,12]. Because the NRF 9.3 is a ratio of nutrients to calories, low energy density foods (e.g., vegetables) scored very high because of their low energy content.

The inclusion of selected food groups makes the NRFh 4:3:3 score more closely related to the HEI-2015 but less closely linked to food cost per calorie. The use of the NRFh score offers a wider range of foods that could be described as both affordable and nutrient-rich. Data in Table 4 suggest that the improvements in diet quality following food replacement modeling could have been achieved without a significant average increase in food cost.

Initial tests of the NRF 9.3 model, conducted some years ago, showed that nutrient density and energy density were inversely linked [11,12]. The relation to energy density was stronger when the model was based on a few micronutrients and was attenuated as more vitamins and minerals were introduced. The one surprise was that adding more vitamins and minerals to the model beyond a certain limit had little additional impact on food-group rankings. In other words, a model based on 23 positive nutrients provided rankings similar to those generated by a model based on 9 or 11 positive nutrients, with correlation levels exceeding 0.90. This is an important consideration, since stakeholders seeking to use nutrient profile models, including regulatory agencies, the food industry, researchers, and health professionals, would most likely prefer a minimal number of nutrients for the ease of use, transparency, and availability of data, whereas models based on an “optimal” number might show higher correlations with a healthy diet.

Past research [28,29] has shown that nutrient-dense foods were associated with higher per calorie costs. Models based primarily on nutrients to limit (fat, sugar, and salt) tend to be highly correlated with energy density [9]. Given that sugar and fat provide dietary energy at low cost, foods deemed “healthy” by many NP models also tend to be more expensive [28]. By contrast, energy-dense foods tend to cost less. Now that affordable nutrient density has become a leading concept, the 2020–2025 *Dietary Guidelines for Americans* stress that “a healthy dietary pattern can be affordable and fit within budgetary constraints” and note that the USDA will be providing an update to their Thrifty Food Plan at the end of 2022 [30]. Identifying those foods that are both nutrient dense and affordable is one way to help implement dietary guidance. Health professionals and policy makers have an interest in supporting food choices and dietary patterns that are both healthy and budget friendly.

Our replacement modeling showed that replacing bottom tertile foods (T1) with more nutrient-rich options (T3) led to significant increases in diet quality as measured using the HEI-2015, and that complete replacement of less nutrient dense foods was not needed to see meaningful increases in the HEI-2015 scores. Statistically significant increases in HEI-2015 were observed with 10–30% replacement, suggesting that even small shifts toward more nutrient-rich foods can have an impact on diet quality.

The study had limitations. First, replacement modeling depends on the selection of food groups and food categories; the present choice was to follow the What We Eat in America scheme. Second, the total cost of the observed and modeled diets was not assessed; such analyses are needed to predict the feasibility of dietary change.

## 5. Conclusions

The NRFh nutrient density score that incorporates both nutrients and desirable food groups was less strongly correlated with food prices per 100 kcal and could be a suitable tool to assess affordable nutrient dense foods. Replacing less nutrient-dense with more nutrient-dense foods, even partially, led to significant improvements in diet quality as measured by HEI-2015.

## Figures and Tables

**Table 1:** Nutrient standards based on Food and Drug Administration values.

Nutrient	Amount	Nutrient	Amount
Protein	50 g	Saturated fat	20 g
Fiber	28 g	Added sugar	50 g
Vitamin A	900 IU	Sodium	2300 mg
Vitamin C	90 mg	Total sugar	90 g
Vitamin D	20 mcg		
Vitamin E	15 mg		
Ca	1300 mg	Dairy	3 cup eq.
Fe	18 mg	Fruit	2 cup eq.
K	4700 mg	Whole grains	3 ounce eq.
Mg	420 mg		
MUFA * + PUFA **	58 g		

* MUFA = monounsaturated fatty acids; ** PUFA = polyunsaturated fatty acids.

**Table 2:** Nutrients, micronutrients, and food groups used in nutrient profiling (NP) models.

NRF * Model	Macronutrients	Vitamins	Minerals	Food Groups	LIM
NRF9.3	Protein, fiber	A, C, D	Ca, Fe, K, Mg	--	Saturated fat, added sugar, sodium
NRF8.3	Protein, fiber	A, C, D	Ca, Fe, K	--	Saturated fat, added sugar, sodium
NRF6.3	Protein, fiber	D	Ca, Fe, K	--	Saturated fat, added sugar, sodium
NRF4.3	Protein, fiber, MUFA + PUFA		K	--	Saturated fat, added sugar, sodium
NRFh 4:3:3	Protein, fiber, MUFA + PUFA		K	Fruit, whole grain, dairy	Saturated fat, added sugar, sodium

* NRF = nutrient-rich food score; Ca calcium, Fe iron, K potassium, Mg magnesium, MUFA mono-unsaturated fatty acids, PUFA poly-unsaturated fatty acids.

**Table 3:** Correlations between NRFh 4:3:3 and NRF n.3 energy density, and food components expressed as %DV.

	NRFh 4:3:3	NRF 4.3	NRF 6.3	NRF 8.3	NRF 9.3
NRFh 4:3:3	--	0.851 **	0.533 **	0.534 **	0.532 **
Energy density (kcal/100 g)	−0.187 **	−0.166 **	−0.377 **	−0.403 **	−0.409 **
Cost per 100 kcal	0.202 **	0.254 **	0.398 **	0.427 **	0.442 **
Cost per RACC	0.121 **	0.218 **	0.032 **	0.046 **	0.044 *
Protein %DV	0.300 **	0.474 **	0.174 **	0.203 **	0.206 **
Fiber %DV	0.351 **	0.380 **	0.655 **	0.654 **	0.672 **
Calcium %DV	0.192 **	0.095 **	0.501 **	0.521 **	0.539 **
Potassium %DV	0.327 **	0.400 **	0.636 **	0.691 **	0.718 **
Vitamin D %DV	0.087 **	0.082 **	0.037 *	0.135 **	0.129 **
Vitamin C %DV	0.253 **	0.212 **	0.751 **	0.745 **	0.734 **
Whole grain %DV	0.292 **	0.049 **	0.014	−0.002	0.007
Fruit %DV	0.491 **	0.133 **	0.185 **	0.178 **	0.168 **
Dairy %DV	0.126 **	−0.084 **	−0.017	0.003	0.005

** Correlation is significant at the 0.02 level; * correlation is significant at the 0.05 level.

**Table 4:** Lowest and highest ranking food categories (tertiles of NRF 9.3 and NRFh 4:3:3) by What We Eat in America 4-digit codes.

Lowest Tertile Of NRF 9.3 Scores	Highest Tertile of NRF 9.3 Scores	Lowest Tertile of NRFh 4:3:3 Scores	Highest Tertile of NRFh 4:3:3 Scores
Apples	Apple juice	Bacon	Apples
Bacon	Beans, peas, legumes	Biscuits, muffins, quick breads	Apple juice
Biscuits, muffins, quick breads	Berries	Butter and animal fats	Bagels and English muffins
Burgers (single code)	Carrots	Cakes and pies	Bananas
Butter and animal fats	Citrus fruits	Candy containing chocolate	Beans, peas, legumes
Cakes and pies	Citrus juice	Candy not containing chocolate	Beef, excludes ground
Candy containing chocolate	Dark green vegetables not lettuce	Cereal bars	Berries
Candy not containing chocolate	Fish	Cold cuts and cured meats	Cheese
Cereal bars	Flavored milk, lowfat	Cookies and brownies	Chicken, whole pieces
Chicken patties, nuggets, tenders	Flavored milk, nonfat	Cream and cream substitutes	Citrus fruits
Cold cuts and cured meats	Flavored milk, reduced fat	Cream cheese, sour cream, whipped cream	Citrus juice
Cookies and brownies	Grits and other cooked cereals	Dips, gravies, other sauces	Crackers, excludes saltines
Crackers, excludes saltines	Lettuce and lettuce salads	Doughnuts, sweet rolls, pastries	Dark green vegetables not lettuce
Cream and cream substitutes	Liver and organ meats	Egg/breakfast sandwiches	Dried fruits
Cream cheese, sour cream, whipped cream	Melons	Frankfurter sandwiches	Fish
Dips, gravies, other sauces	Milk substitutes	Frankfurters	Flavored milk, lowfat
Doughnuts, sweet rolls, pastries	Milk, lowfat	Gelatins, ices, sorbets	Flavored milk, nonfat
Egg/breakfast sandwiches	Milk, nonfat	Ice cream, frozen dairy desserts	Grapes
Frankfurter sandwiches	Milk, reduced fat	Jams, syrups, toppings	Lamb, goat, game
Frankfurters	Milk, whole	Macaroni and cheese	Lettuce and lettuce salads
French fries/fried white potatoes	Mustard and other condiments	Margarine	Melons
Gelatins, ices, sorbets	Nutrition bars	Mayonnaise	Milk, lowfat
Ice cream, frozen dairy desserts	Nutritional beverages	Milk shakes, other dairy drinks	Milk, nonfat
Jams, syrups, toppings	Oatmeal	Pretzels/snack mix	Milk, reduced fat
Macaroni and cheese	Olives, pickles, pickled vegetables	Pudding	Milk, whole
Margarine	Onions	Salad dressings, vegetable oils	Nutritional beverages
Mayonnaise	Other fruit juice	Sausages	Nuts and seeds
Milk shakes, other dairy drinks	Other fruits and fruit salads	Soft drinks	Oatmeal
Peanut butter and jelly sandwiches	Other red and orange vegetables	Soy-based condiments	Other fruit juice
Popcorn	Other starchy vegetables	Sport and energy drinks	Other fruits and fruit salads
Pretzels/snack mix	Other vegetables and combos	Sugar substitutes	Other starchy vegetables
Pudding	Peaches and nectarines	Sugars and honey	Pasta, noodles, cooked grains
Rice	Processed soy products	Tea	Peaches and nectarines
Salad dressings, vegetable oils	RTE cereal, higher sugar (>21.2 g/100 g)	Tomato-based condiments	Popcorn
Saltine crackers	RTE cereal, lower sugar (≤21.2 g/100 g)	Turnovers, grain-based items	Processed soy products
Sausages	Shellfish	Coffee	RTE cereal, lower sugar (≤21.2 g/100 g)
Soft drinks	Smoothies and grain drinks	Egg rolls, dumplings, sushi	Smoothies and grain drinks
Soy-based condiments	String beans	Fruit drinks	Tomatoes
Sport and energy drinks	Tomatoes	Mashed potatoes, white potato mixture	Tortilla, corn, other chips
Sugar substitutes	Vegetable juice	Pasta sauces, tomato-based	Turkey, duck, other poultry
Sugars and honey	Vegetable mixed dishes	Soups	Yeast breads
Tea	White potatoes, baked or boiled	Milk substitutes	Vegetable juice
Tomato-based condiments	Yogurt, Greek	Olives, pickles, pickled vegetables	Yogurt, Greek
Turnovers, grain-based items	Yogurt, regular	RTE cereal, higher sugar (>21.2 g/100 g	Yogurt, regular
			
mean cost USD 0.29/100 kcal	mean cost USD 0.70/100 kcal	mean cost USD 0.37/100 kcal	mean cost USD 0.58/100 kcal

**Table 5:** Effect of replacing nutrient density T1 foods with T3 foods at 10%, 20%, 30%, and 100% level on HEI-2015 scores by gender and age group.

Gender	Age (y)	n	Percent Replacement	NRF 9.3			NRFh 4:3:3		
				Change in HEI-2015 ± SEM	Lower 95th CI	Upper 95th CI	Change in HEI-2015 ± SEM	Lower 95th CI	Upper 95th CI
Male	2–18	2856	10%	2.75 ± 0.15 *	2.45	3.06	3.28 ± 0.16 *	2.96	3.60
			20%	4.04 ± 0.22 *	3.60	4.48	4.89 ± 0.22 *	4.43	5.34
			30%	4.75 ± 0.27 *	4.21	5.30	5.77 ± 0.27 *	5.23	6.32
			100%	5.47 ± 0.38 *	4.70	6.24	6.81 ± 0.39 *	6.02	7.60
	19+	4955	10%	3.41 ± 0.11 *^	2.51	2.92	4.14 ± 0.10 *	3.03	3.41
			20%	4.75 ± 0.17 *^	3.71	4.28	5.77 ± 0.15 *	4.54	5.10
			30%	5.38 ± 0.20 *^	4.36	5.05	6.53 ± 0.18 *	5.37	6.08
			100%	5.56 ± 0.30 *^	4.79	5.87	6.96 ± 0.26 *	6.18	7.28
Female	2–18	2813	10%	2.71 ± 0.10 *^	3.17	3.64	3.22 ± 0.09 *	3.92	4.35
			20%	3.99 ± 0.14 *^	4.41	5.09	4.82 ± 0.14 *	5.46	6.07
			30%	4.70 ± 0.17 *^	4.97	5.79	5.73 ± 0.17 *	6.17	6.90
			100%	5.33 ± 0.27 *^	4.95	6.18	6.73 ± 0.27 *	6.43	7.48
	19+	5157	10%	3.31 ± 0.09 *^	3.12	3.50	4.01 ± 0.09 *	3.83	4.20
			20%	4.65 ± 0.14 *^	4.37	4.93	5.67 ± 0.15 *	5.37	5.97
			30%	5.26 ± 0.17 *^	4.92	5.61	6.45 ± 0.19 *	6.07	6.83
			100%	5.37 ± 0.28 *^	4.79	5.95	6.77 ± 0.31 *	6.14	7.41

* Indicates change in HEI-2015 total score is significantly different from zero, *p* < 0.05 ^ Indicates non-overlapping 95th percentile confidence intervals of change in HEI-2015 total score between NRF 9.3 and HNDS 4.3.3.

**Table 6:** Effect of replacing nutrient density T1 foods with T3 foods at 10%, 20%, 30%, and 100% level on HEI-2015 scores among adults (age > 19 years) by gender and PIR group.

Gender	PIR	n	Percent Replacement	NRF 9.3			NRFh 4:3:3		
				Change in HEI-2015 ± SEM	Lower 95th CI	Upper 95th CI	Change in HEI-2015 ± SEM	Lower 95th CI	Upper 95th CI
Male	<1.35	1467	10%	2.66 ± 0.16 *^	2.32	2.99	3.36 ± 0.16 *	3.04	3.69
			20%	3.88 ± 0.23 *^	3.40	4.35	4.90 ± 0.24 *	4.41	5.39
			30%	4.54 ± 0.29 *^	3.95	5.12	5.76 ± 0.30 *	5.15	6.37
			100%	5.14 ± 0.45 *	4.22	6.06	6.80 ± 0.43 *	5.93	7.66
	1.35–1.85	563	10%	2.79 ± 0.29 *	2.19	3.38	3.74 ± 0.34 *	3.05	4.43
			20%	3.71 ± 0.43 *	2.84	4.58	5.27 ± 0.49 *	4.27	6.27
			30%	4.16 ± 0.51 *	3.11	5.20	6.02 ± 0.59 *	4.82	7.21
			100%	4.36 ± 0.69 *	2.96	5.76	6.69 ± 0.78 *	5.11	8.27
	>1.85	2521	10%	3.32 ± 0.14 *^	3.04	3.60	4.06 ± 0.14 *	3.77	4.34
			20%	4.63 ± 0.20 *^	4.21	5.05	5.64 ± 0.20 *	5.22	6.05
			30%	5.19 ± 0.26 *^	4.67	5.71	6.31 ± 0.24 *	5.82	6.81
			100%	5.10 ± 0.39 *	4.32	5.89	6.48 ± 0.35 *	5.76	7.19
Female	<1.35	1683	10%	3.03 ± 0.14 *	2.74	3.32	3.63 ± 0.18 *	3.27	3.99
			20%	4.48 ± 0.20 *	4.06	4.90	5.37 ± 0.26 *	4.85	5.90
			30%	5.30 ± 0.25 *	4.78	5.81	6.33 ± 0.31 *	5.70	6.95
			100%	6.36 ± 0.42 *	5.51	7.22	7.58 ± 0.45 *	6.66	8.51
	1.35–1.85	557	10%	3.14 ± 0.35 *	2.43	3.85	3.84 ± 0.32 *	3.19	4.49
			20%	4.44 ± 0.51 *	3.41	5.47	5.42 ± 0.48 *	4.44	6.41
			30%	5.06 ± 0.60 *	3.84	6.28	6.16 ± 0.57 *	5.00	7.33
			100%	5.11 ± 0.78 *	3.51	6.70	6.37 ± 0.80 *	4.75	8.00
	>1.85	2476	10%	3.69 ± 0.15 *	3.38	4.00	4.07 ± 0.13 *	3.79	4.34
			20%	5.04 ± 0.23 *	4.57	5.50	5.60 ± 0.22 *	5.15	6.05
			30%	5.52 ± 0.28 *	4.95	6.09	6.25 ± 0.28 *	5.68	6.82
			100%	5.00 ± 0.42 *	4.15	5.86	6.08 ± 0.45 *	5.16	7.01

* Indicates change in HEI0-2015 total score is significantly different from zero, *p* < 0.05. ^ Indicates non-overlapping 95th percentile confidence intervals of change in HEI-2015 total score between NRF 9.3 and HNDS 4.3.3.

## Data Availability

All NHANES data are publicly available on the NCHS and USDA websites. All documentation is provided online at https://www.cdc.gov/nchs/nhanes/index.htm (accessed on 19 May 2021).
